# Influence of six different RE^3+^ ions as modifier agents on the photoluminescent, electrical, magnetic and thermal properties of B-Na glass

**DOI:** 10.1038/s41598-026-35015-5

**Published:** 2026-02-04

**Authors:** M. M. El-shabaan, Amaal Mohamed, M. I. Youssif, N. A. El-Ghamaz, E. M. Ahmed

**Affiliations:** 1 Physics Department, Faculty of Science, Damietta University, New Damietta, 34517 Egypt; 2 Supportive Requirement Department, College of Engineering and Technology, University of Technology and Applied Sciences, Suhar, 311 Oman

**Keywords:** Materials science, Physics

## Abstract

This study deals with investigation of the influence of rare-earth (RE³⁺) ions La³⁺, Nd³⁺, Gd³⁺, Ho³⁺, Er³⁺, and Yb³⁺ on the structural, photoluminescent, electrical, and thermal properties of a simple 50%B₂O₃ − 50%Na₂O glass system. The incorporation of 1 mol% RE₂O₃ systematically enhanced optical polarizability, as indicated by the increase in molar refraction (*R*_*m*_) and nonlinear susceptibility (*χ⁽³⁾*). The Er³⁺ doped glass is exhibiting the highest value of *χ⁽³⁾* ≈ 1.76 × 10⁻¹² esu. The calculated optical basicity, oxide ion polarizability, and metallization criteria confirmed the nonmetallic nature of all compositions. RE addition markedly intensified the photoluminescence emission, particularly for Gd³⁺ (> 2000 a.u.) and Er³⁺ (~ 700 a.u.), accompanied by high correlated color temperature values (*CCT* > 7600 K). Thermal analyses (TGA/DSC) revealed excellent stability up to 800 °C, with *T*_*g*_ values increasing from 422 °C (base glass) to 450 °C depending on RE type; the Nd³⁺-doped glass showed the highest thermal stability (*ΔT* ≈ 120 °C), implying superior glass-forming ability. All RE-doped samples displayed paramagnetic behavior, except La³⁺-doped, which remained diamagnetic. The dc conductivity decreased with decreasing ionic radius, consistent with the correlated barrier hopping (CBH) mechanism, while thermal conductivity (0.49–1.78 W m⁻¹ K⁻¹) confirmed their insulating and thermoelectric potential. Overall, the results demonstrate that RE³⁺ ions effectively tailor multifunctional characteristics of sodium borate glasses, highlighting their promise for advanced photonic and energy-related applications.

## Introduction

In recent years, the development of advanced functional glasses has accelerated due to growing demands in photonics, optoelectronics, biomedical devices, energy systems, and radiation shielding technologies. Among the various glass families, borate-based glasses have emerged as versatile host matrices because of their low melting temperatures, broad compositional flexibility, high rare-earth (RE) solubility, and favorable optical transmission in the ultraviolet and visible regions^1–3^. These attributes make borate glasses particularly attractive for solid-state lasers, luminescent devices, and thermally stable protective coatings.

Boron oxide (B₂O₃) acts as a classical network former in such systems. Unlike silicate networks dominated by tetrahedral coordination, boron atoms can adopt both trigonal ([BO₃]) and tetrahedral ([BO₄]) units, imparting exceptional structural adaptability. This hybrid coordination is sensitive to the addition of alkali oxides (e.g., Na₂O), which act as network modifiers that depolymerize the B–O–B network, reduce viscosity and melting temperature, and influence the glass transition (*T*_*g*_) and crystallization (*T*_*c*_) behaviors^4–6^.

Incorporating rare-earth ions (RE³⁺) into borate glasses has attracted considerable attention because of their unique 4*f* electronic configurations, large ionic field strengths, and high coordination tendencies. RE ions such as La³⁺, Nd³⁺, Gd³⁺, Ho³⁺, Er³⁺, and Yb³⁺ can act as both modifiers and intermediate species depending on their ionic radius and charge-to-radius ratio, thereby altering the connectivity of the borate network and influencing optical and thermal responses^7–10^. Previous studies have shown that smaller RE ions (e.g., Er³⁺, Yb³⁺) increase *T*_*g*_ through stronger RE–O bonding and enhanced network crosslinking, while larger RE ions (e.g., La³⁺) tend to weaken the network due to their lower field strength^11–14^. The resulting variations in *T*_*g*_, *T*_*c*_, and thermal stability index (ΔT) reflect how RE ion size and bonding nature govern the glass-forming ability (GFA).

Recent investigations further highlight that the mismatch in ionic radius, coordination flexibility, and bond enthalpy among RE ions strongly affects the structural stability and crystallization resistance of borate glasses^15–17^. Furthermore, recent research has emphasized the role of ionic radius mismatch, coordination flexibility and bond enthalpy in governing the thermal stability and glass-forming ability of RE-doped systems^18,19^. Studies employing molecular dynamics simulations and advanced spectroscopic methods (e.g., FTIR, Raman and EXAFS) have confirmed that RE ions can act both as network modifiers and intermediate species, depending on their charge-to-radius ratio and local environment^20–22^. Such structural versatility enables precise control of optical and thermal functionalities, making RE-doped borate glasses suitable for applications in high-temperature sensors, scintillators, and photothermal converters.

Despite these advances, most earlier studies have focused on individual RE oxides or limited compositional ranges, leaving a lack of comprehensive comparative investigations within a single base glass system. Therefore, the present work systematically examines the effect of six different RE₂O₃ dopants (La₂O₃, Nd₂O₃, Gd₂O₃, Ho₂O₃, Er₂O₃, and Yb₂O₃) on the structural, photoluminescent, electrical, magnetic, and thermal characteristics of the simple 50B₂O₃–(50–x)Na₂O–xRE₂O₃ (x = 1 mol%) glass series. This unified approach provides a direct correlation between 4*f*-electron configuration, ionic radius, and multifunctional material properties under identical preparation conditions.

The main objectives of this study are to (i) elucidate how RE ionic type modulates the composition - physical property relationships in sodium borate glass, (ii) identify trends linking 4*f*-electron filling to optical, electrical, and thermal behavior, and (iii) determine the most promising RE dopant for potential photonic and thermoelectric applications. This comparative methodology distinguishes the present work from prior single-ion studies and contributes valuable insight into the design of next-generation multifunctional borate glasses.

## Experimental details

The melt quenching technique is used to prepare glasses having the composition 50%B₂O₃(50- x)%Na₂O-x%RE₂O₃, where (x = 0 and 1) and (RE = La, Nd, Gd, Ho, Er and Yb). Analytical-grade chemicals were employed for glass preparation, including boric acid (H₃BO₃, 99.9%,Sigma-Aldrich), sodium carbonate (Na₂CO₃, 99.9%, Sigma-Aldrich), and the respective rare-earth oxides (RE₂O₃, ≥ 99.99%, Sigma-Aldrich). The weighed batches (≈ 20 g each) were measured with an accuracy of ± 0.0001 g using an electronic balance. The raw powders were thoroughly mixed in an agate mortar for at least 15 min to ensure compositional homogeneity. Melting was carried out in an electric furnace at 1000 °C for 1 h under ambient atmosphere, with intermittent stirring using a platinum rod to promote uniform mixing. The resulting melts were rapidly quenched by pouring onto a preheated stainless-steel plate and immediately pressed with another plate to form flat glass pieces. The quenched samples were subsequently annealed at 400 °C for 3 h to remove residual thermal stresses and then slowly cooled to room temperature inside the furnace.The optical images of all studied glass samples are depicted in Table 1. The photoluminescence emission spectra are recorded using JASCO FP-8350 spectrofluorometer for excitation and emission in the 400–750 nm wavelength range using λ_ex_ = 220 nm is made via monochromator. Pieces of the glassy samples are well polished and painted with silver paste to achieve good ohmic contact electrodes for electrical measurements. *σ*_*dc*_ is carried out using Keithley model 6517b electrometer. A magnetic susceptibility balance of type Sherwood MSB MK1, Cambridge UK, is used to explore the magnetic characteristics of the tested glasses at room temperature. TGA-DTA measurements were carried out on a TA Instruments SDT Q600 V20.9 Build 20 instrument under nitrogen flow, heating from room temperature to 800 °C, with a heating rate of 10 °C/min. Thermal conductivity measurement is made by using Lee’s disc technique with homemade setup.

**Table 1:** The optical images of all studied glass samples.

Sample code	B-Na	B-Na-La	B-Na-Nd	B-Na-Gd	B-Na-Ho	B-Na-Er	B-Na-Yb
Sample image							

## Results and discussion

### Metallization criterion (*M*)

A crucial parameter for predicting the insulation behavior and the ability of the fabricated glasses for metallization is the Metallization Criterion (*M*). Values of *M* can be derived from the following relation^23^:

1 $$\:M=1-\frac{{R}_{m}}{{V}_{m}}$$

where *R*_*m*_ is the molar refraction and $\:{V}_{m}$ is the molar volume of the glasses. *R*_*m*_ of the investigated glasses correlates with the refractive index *n* and the molar volume $\:{V}_{m}$ through the following expression^23^:

2 $$\:{R}_{m}=\frac{({n}^{2}-1)}{({n}^{2}+2)}{V}_{m}$$

The molar polarizability, $\:{\alpha\:}_{m}$, is found to correlate to *R*_*m*_ values by the following Eq. 2^3^:

3 $$\:{\alpha\:}_{m}=\frac{3}{4\pi\:{N}_{A}}{R}_{m}=\frac{{R}_{m}}{2\cdot \:52}$$

where *N*_*A*_ is the Avogadro’s number. According to Herzfeld^24^, a material is deemed to exhibit metallic properties if $\:{R}_{m}/{V}_{m}>\:1$, and non-metallic if $\:{R}_{m}/{V}_{m}<\:1$. Values of n and $\:{V}_{m}$ which are used in the calculations are collected from Ref.^25^. The obtained values of $\:{R}_{m}$, $\:{\alpha\:}_{m}$ and *M* are presented in Table 2. It is observed that, $\:{R}_{m}$ and $\:{\alpha\:}_{m}$ of the RE doped samples have higher values than the values of the RE free sample. Doping the glass matrix with RE ions results in an increase in the number of NBOs, thereby augmenting the population of free electrons in the glass system. Consequently, the values of $\:{R}_{m}$ and $\:{\alpha\:}_{m}$ increase^26^. According to the results of *M* presented in Table 2, the positive values of *M* (in the range 0.396–0.412) confirms the non-metallic behavior of the studied glasses. The slight decrease in *M* upon doping with Ho^3+^ and Er^3+^ signifies a broadening of both the valence and conduction bands which leads to a decrease of *E*_*g*_^23^.

**Table 2:** Molar refraction (*R*_*m*_), molar polarizability (*α*_*m*_), metallization criterion (*M*), optical basicity, single bond strength (*B*_*M−O*_), interaction parameter (A^*^), and 3rd order nonlinear optical susceptibility (*χ*^*(3)*^) of the studied glasses.

Sample	*R*_m_ (cm^3^/mol)	α_m_ (Å^3^)	M	Λ_th_	Λ_exp_	B_M−O_ (kJ/mol)	A^*^ (Å^−3^)	χ^(3)^ × 10^− 12^ (esu)
B-Na	16.12	6.39	0.407	0.78750	1.132772	291	0.0325	1.47
B-Na-La	16.30	6.47	0.410	0.78550	1.131696	298.15	0.0326	1.39
B-Na-Nd	16.38	6.50	0.410	0.78614	1.134562	297.19	0.0317	1.40
B-Na-Gd	16.40	6.51	0.409	0.78569	1.135686	297.32	0.0314	1.43
B-Na-Ho	16.58	6.58	0.402	0.78920	1.141864	296.08	0.0297	1.58
B-Na-Er	16.76	6.65	0.396	0.78920	1.147529	296.31	0.0280	1.76
B-Na-Yb	16.33	6.48	0.412	0.78493	1.133422	294.139	0.0321	1.36

### Optical basicity and oxide ion polarizability

The optical basicity and average electronic polarizability functions are found closely linked to the glass’s usefulness in the optical and electronic applications. Optical basicity, $\:\varLambda\:$, quantifies the electron density carried by oxygen. It has been established that, the optical non-linearity arises from the electronic polarization of a material under exposure intense of light. Consequently, materials with heightened optical non-linearity need to be identified or engineered based on the correlation between optical non-linearity and other readily understandable electronic properties^27^. The concept of $\:\varLambda\:$, hinges strongly on the bonding nature (ionic or covalent) within the glass network. The theoretical optical basicity $\:{\varLambda\:}_{th}$ is computed using the following equation^23^:

4 $$\:{\varLambda\:}_{th}=\sum\:_{i}{X}_{i}{\varLambda\:}_{i}$$

where $\:{X}_{i}\:$represents the molar fraction of the oxide in the glass, and $\:{\varLambda\:}_{i}$ denotes the optical basicity of the individual oxides listed in Table 3. As per Duffy and Ingram^28^, Eq. 4 aims to predict trends in optical basicity rather than providing an absolute value. However, since this equation fails to consider changes in cation coordination numbers, it cannot accurately estimate basicity values. Therefore, it’s crucial to assess the experimental optical basicity $\:{\varLambda\:}_{exp}$ through the following relationship^23^:

**Table 3:** The values of the optical basicity (*Λ*_*i*_) of individual oxides, and the molar polarizability *α*_*i*_ of their corresponding cations.

Oxide	Λ_i_	α_i_ (Å^3^)	Ref.
B_2_O_3_	0.425	0.002	^23,115^
Na_2_O	1.150	0.175	^116^
La_2_O	1.048	1.32	^117^
Nd_2_O_3_	1.014	1.25	^117^
Gd_2_O_3_	0.969	1.08	^117^
Ho_2_O_3_	0.945	0.91	^117^
Er_2_O_3_	0.929	0.89	^117^
Yb_2_O_3_	0.893	0.86	^117^

5 $$\:{\varLambda\:}_{{exp}_{\cdot \:}}=1.67\left(1-\frac{1}{{\alpha\:}_{{O}^{2-}}}\right)$$

where $\:{\alpha\:}_{{O}^{2-}}$ is the oxide ion polarizability which is related to the molar polarizability by the relation:

6 $$\:{\alpha\:}_{m}=N{\alpha\:}_{{O}^{2-}}+\sum\:{\alpha\:}_{i}$$

where *N* represents the number of oxide ions in the glass formula, and *∑α*_*i*_ signifies the molar cation polarizability which can be represented as follows:

7 $$\:\sum\:{\alpha\:}_{i}=\sum\:\left({c}_{i}{\alpha\:}_{i}\right)$$

where *c*_*i*_ and *α*_*i*_ are the molar fraction and the polarizability of the cation (i), respectively. The values of *c*_*i*_ and *α*_*i*_ of the individual cations are listed in Table 3. The obtained values of $\:{\varLambda\:}_{th}$.and $\:{\varLambda\:}_{exp}$ are displayed in Table 2 for comparison. The base glass composition (50%B_2_O_3_-50%Na_2_O) exhibits a value of $\:{\varLambda\:}_{exp}$ ≈ 1.133, and a value of $\:{\varLambda\:}_{th}$ ≈ 0.788. Introducing 1% of various RE oxides (La_2_O_3_, Nd_2_O_3_, Gd_2_O_3_, Ho_2_O_3_, Er_2_O_3_, or Yb_2_O_3_) at the expense of Na_2_O modifies these properties, with the effects varying by RE type due to differences in their ionic radii, polarizabilities, and basicities. These changes reflect how RE ions influence the electron donor ability of oxide ions and interionic interactions in the borate network. $\:{\varLambda\:}_{th}$ shows minor variations, ranging from 0.785 (Yb_2_O_3_) to 0.789 (Ho_2_O_3_ and Er_2_O_3_). This trend suggests that the assigned basicity values for heavier RE oxides like Ho and Er are higher than for lighter ions like La and Yb, leading to a trivial enhancement in the average electron donor power when these are incorporated. The small shifts align with the low doping level and the compositional averaging in theoretical calculations. On the other hand, the trend of $\:{\varLambda\:}_{exp}$ is found generally increases with atomic number across the lanthanides (La < Nd < Gd < Ho < Er), except for Yb, which dips back closer to the base glass. This indicates that heavier RE ions with smaller ionic radii enhance the electron donor power more effectively, possibly by altering the borate network structure, increasing non-bridging oxygens, or enhancing ion polarizability^25^.

Across all compositions, $\:{\varLambda\:}_{exp}$ (1.132–1.148) is consistently higher than $\:{\varLambda\:}_{th}$ (0.785–0.789) by approximately 0.34–0.36. This significant gap is suggested to arise primarily from limitations in the theoretical model and structural complexities in borate glasses^26^. In borate glasses, boron undergoes a coordination shift from trigonal (BO_3_) to tetrahedral (BO_4_) with alkali addition. This transition involves a loss of π-bonding in BO_4_ units, which increases the polarizing power of boron and enhances the overall basicity beyond what the theoretical model predicts. Additionally, from Eqs. (4)–(7), theoretical optical basicity $\:{\varLambda\:}_{th}$ is calculated as a weighted average of the basicities of individual oxides, assuming ideal additive behavior without accounting for structural rearrangements. In contrast, experimental optical basicity $\:{\varLambda\:}_{exp}$ is typically dependent on their calculation on several sensitive factors such as, oxide ion and molar cation polarizabilities. Furthermore, it is noticed that $\:{\varLambda\:}_{exp}$ has the same trend as that of refractive index (n) upon doping with different RE-ions. The increase in $\:{\varLambda\:}_{exp}$ values may be explained by the decrease in ionic radius across the RE series (from La³^+^ to heavier elements like Nd³^+^, Gd³^+^, Ho³^+^, and Er³⁺), leading to higher polarizability. These ions can increase the refractive index by encouraging more covalent bonding with the oxygen atoms in the glass network, thereby increasing the optical basicity. Furthermore, Yb³⁺ has one of the smallest ionic radii among the RE-ions, it possesses fewer unpaired electrons and lower polarizability compared to other RE-ions, and consequently, this reduces its ability to increase the refractive index and optical basicity^29–31^. According to previous discussion for the correlation between the structural and optical properties. The glasses under investigation have proved to be good candidates for optical application as well as photonic materials, and laser applications^29–31^.

### Average single bond strength of the glasses (*B*_*M─O*_)

Drawing on Sun’s basic principle of glass formation^32^, Dimitrov et al.^33^ introduced a straightforward approach to determine the average single bond strength (B_M─O_) of oxide glasses. The B_M─O_ expression for the glass system being studied can be formulated as follows:

8 $$\:{B}_{M-O}={xB}_{B-O}+{yB}_{Na-O}+{zB}_{RE-O}$$

where *B*_*B-O*_, *B*_*Na-O*_, and *B*_*RE-O*_, are the single bond strength of the corresponding oxides, and the coefficients *x*, *y*, and *z* are the molar percent of each oxide in the glass composition. Single bond strength of the corresponding individual oxides are taken as 498, 84, 799, 703, 716, 592, 615, and 397.9 kJ/mol for B-O, Na-O, La-O, Nd-O, Gd-O, Ho-O, Er-O, and Yb-O, respectively^34,35^. The calculated *B*_*M*─*O*_ value of the glasses under test is presented in Table 2. The B-Na sample exhibits a lowest *B*_*M*─*O*_ value of 291 kJ/mol, due to the presence of Na⁺ ions, which act as network modifiers by breaking B–O–B linkages and creating NBOs, thereby weakening the glass network^25^. Upon substituting 1 mol% of Na₂O with 1 mol% of the different RE-oxides, the bond strength is found to increase. A maximum value of 298.15 kJ/mol for the B-Na-La glass is noticed. This can be attributed to the higher field strength and trivalent charge of La³⁺, which enhance B–O–RE linkages and consequently, contribute to a more compact and stronger network, despite the La³⁺ have a larger ionic radius^36,37^. A slight decrease in *B*_*M*─*O*_ values is observed with Nd³⁺ (297.19 kJ/mol) and Gd³⁺ (297.32 kJ/mol), likely due to their moderate ionic sizes and field strengths. The further decrease with Ho³⁺ (296.08 kJ/mol), Er³⁺ (296.31 kJ/mol), and especially Yb³⁺ (294.14 kJ/mol) can be explained by increase of network distortion and NBO formation, particularly for Yb³⁺ whose filled 4*f* shell ([Xe]4*f*¹⁴) reduces bonding interactions with the glass matrix. These trends are consistent with the previously obtained results for FTIR and *N₄* structural data, which show a decrease in BO₄ unit content with certain RE ions, reflecting a direct relationship between bond strength and network structure^25^.

### Interaction parameter (A^*^)

The extent of charge overlap between oxide ions and their nearest neighboring cations are quantified by the interaction parameter (A^*^), which can be evaluated using the method introduced by Dimitrov et al.^38,39^. For the investigated glasses, the parameter A^*^ is determined via Eq. 9, and presented in Table 2.

9 $$\begin{aligned} \:A^{*} & = X_{{B_{2} O_{3} }} \frac{{\left( {\alpha \:_{f}^{ - } - \alpha \:_{{O^{{2 - }} }} } \right)}}{{2(\alpha \:_{{B^{{3 + }} }} + \alpha \:_{f}^{ - } )(\alpha \:_{{O^{{2 - }} }} + \alpha \:_{{B^{{3 + }} }} )}} + X_{{Na_{2} O}} \frac{{\left( {\alpha \:_{f}^{ - } - \alpha \:_{{O^{{2 - }} }} } \right)}}{{2(\alpha \:_{{Na^{{2 + }} }} + \alpha \:_{f}^{ - } )(\alpha \:_{{O^{{2 - }} }} + \alpha \:_{{Na^{{2 + }} }} )}} \\ & + X_{{RE_{2} O_{3} }} \frac{{\left( {\alpha \:_{f}^{ - } - \alpha \:_{{O^{{2 - }} }} } \right)}}{{2(\alpha \:_{{RE^{{3 + }} }} + \alpha \:_{f}^{ - } )(\alpha \:_{{O^{{2 - }} }} + \alpha \:_{{RE^{{3 + }} }} )}} \\ \end{aligned}$$

where $\:{\alpha\:}_{f}^{-}$ is the electronic polarizability of the free oxide ion (taken as 3.921 Å^3^)^40^. Furthermore, the value of A^*^ reflects the strength of cation–oxygen bonding and its influence on glass network connectivity and the degree of polarization in the oxide network. The interaction parameter in these glasses, ranging from 0.028 to 0.033 Å^-[3^. A lower $\:{A}^{*}$ value indicates weaker interionic interactions, more ionic bonding, higher unshared electron density on O^2-^ ions, and thus greater optical basicity. The RE free glass sample is found to present the lowest value of A^*^ ≈ 0.0325 Å^- 3^, typical for borate systems known for relatively low interaction. RE doping to the glass generally lowers A^*^ values, reflecting enhanced ionic character and basicity. The A^*^ values are found to present an opposite trend to the $\:{\varLambda\:}_{exp}$: higher basicity correlates with lower interaction, as weaker interactions allow oxide ions to donate electrons more freely. Conversely, La-doped (highest A^*^) shows the least enhancement in basicity. Values of the A^*^ may be affected by the RE-specific polarizabilities and field strengths^25^; smaller ions disrupt the network more, reducing interaction strength. The value of A^*^ is found to slightly decrease upon introducing RE ions to the glass matrix. This trend of A^*^ can be correlated with their number of unpaired 4f electrons which affect both ionic polarizability and bonding characteristics. As the number of unpaired electrons increases across the RE series (La³⁺ to Yb³⁺), the shielding effect of the 4f electrons modulates the effective interaction between RE ions and oxygen. Ions with a higher number of unpaired electrons typically exhibit stronger polarizability and reduced localized fields^25^, leading to subtle reductions in A^*^, as seen for the glasses doped with La³⁺ to Er³⁺. Conversely, deviations from this trend, such as the higher (A^*^) of Yb³⁺ doped sample, suggests that factors like covalency and local structural arrangements also contribute significantly to the increase of the value of A^*^. These findings align with many established theories on the influence of unpaired 4f electrons on glass network modification and polarizability trends^29–38^.

Overall, the low A^*^ values confirm the glasses’ high basicity, with RE type modulating this through polarization effects, making them suitable for optical applications where high electron donor power is desirable^38,41^. These effects highlight a progression toward higher basicity and lower interaction with heavier RE, likely due to decreasing ionic radii and increasing field strengths, which modify the glass’s covalent-ionic balance.

### Third order nonlinear optical susceptibility

Third order nonlinear optical susceptibility (*χ*^(^³^)^) is a fundamental parameter that characterizes a material’s response to intense electromagnetic fields, enabling phenomena such as third-harmonic generation, self-focusing, and optical Kerr effects. It plays a crucial role in the development of advanced photonic devices for optical switching, signal processing, and telecommunications^42,43^. Third order nonlinear susceptibility, *χ*^(3)^, of studied glasses is estimated by the following Miller’s rule^44^:

10 $$\:{\chi\:}^{\left(3\right)}={\left[\frac{{n}^{2}-1}{4\pi\:}\right]}^{4}\times\:{10}^{-10}esu$$

The obtained results of *χ*⁽³⁾ for the studied glasses are presented in Table 2. It is noticed that, a slight decrease in *χ*⁽³⁾ value from 1.47 × 10^–12^ to 1.39 × 10^–12^ esu is recorded upon doping B-Na glass with La³⁺ followed by a continuous increase of *χ*⁽³⁾ upon doping with RE with the order (Nd, Gd, Ho and Er). The continuous reduction of the RE’s ionic radius with high field strength ions is found significantly enhancing the value of *χ*⁽³⁾ due to increased local electric fields and improved network interaction. The maximum magnitude of *χ*⁽³⁾ is recorded for the sample doped with Er³⁺ suggests optimal nonlinear response. The drop in *χ*⁽³⁾ for B-Na-Yb to 1.36 × 10^–12^ esu may be resulted from the structural saturation or RE clustering^45,46^. The studied glasses, especially those doped with Ho³⁺ and Er³⁺, are found to be promising candidates for nonlinear optical applications such as optical switching, frequency conversion, ultrafast signal processing, third-harmonic generation systems, telecommunication devices, and laser amplifiers, where high *χ*⁽³⁾ is essential for efficient performance^47,48^.

### Photoluminescence properties

#### Electronic transitions

**Fig. 1:**
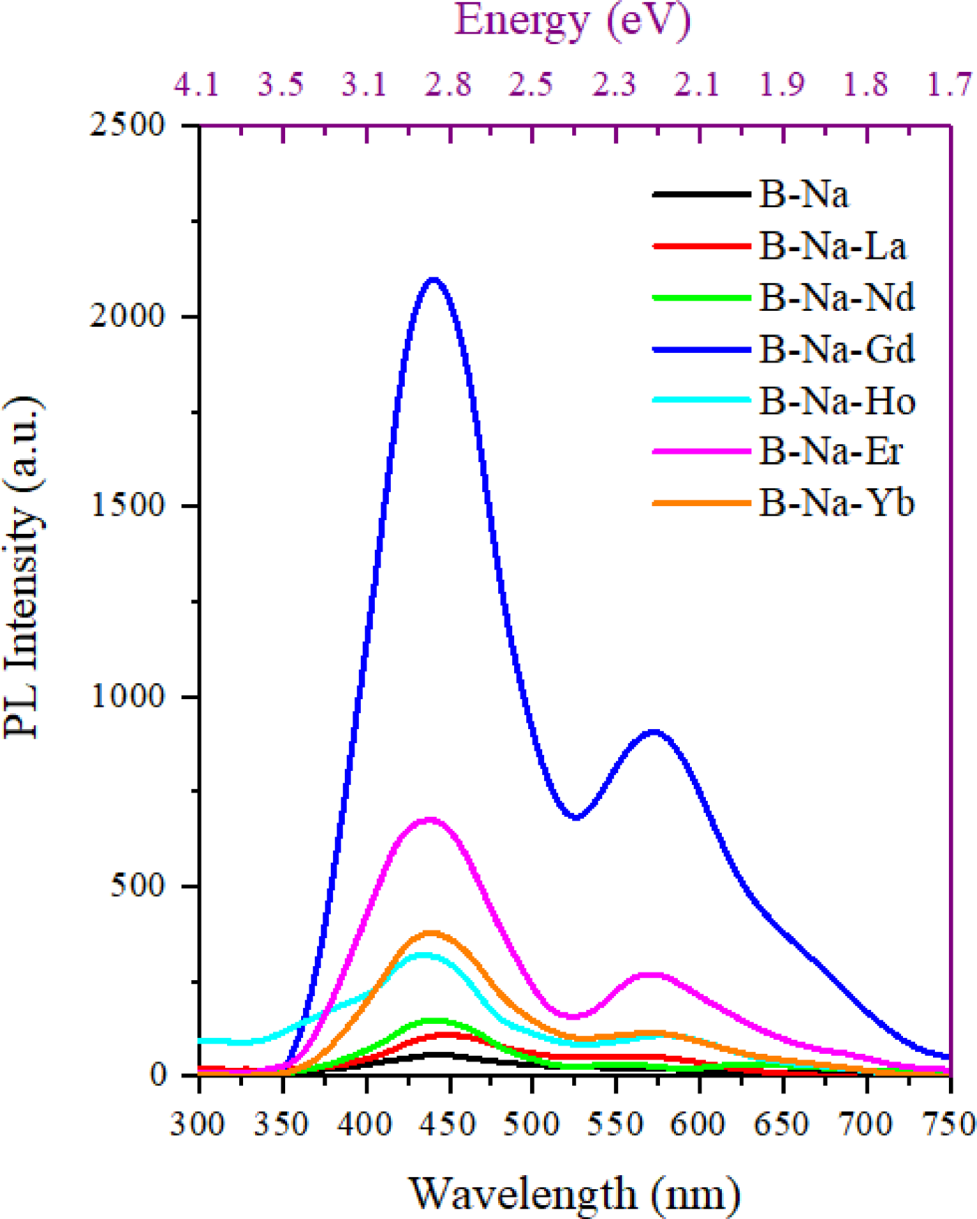
The emission spectra of the glasses doped with different RE_2_O_3_.

Figure 1 displays the emission photoluminescence (PL) spectrum of the glasses with the composition 50%B₂O₃-(50-x)%Na₂O-x%RE₂O₃, (x = 0 and 1) in the wavelength range 300–750 nm. All the glass samples exhibited a dominant characteristic emission peak at around 440 nm (2.82 eV). This peak may be attributed to the intrinsic defects or self-trapped excitons within the borate glass network^49,50^. It is worth noting that B-Na base glass did not present any other additional emission peaks. The weak emission of the B-Na base glass suggests that the glass matrix lacks strong emitting centers. The minor signal may arise from defects or impurities within the borate network, such as boron-oxygen hole centers or NBO defects, which are common in borate-based glasses^51^. The RE-doped glasses exhibit distinct emission profiles depending on the RE element with various degrees of enhanced intensity compared to the undoped glass. The spectrum of the La doped B-Na glass shows a slight increase of the emission intensity (below 120 a.u.) peaking around 457 nm, with a secondary rise at 567 nm which may be corresponding to the ^4^G_5/2_ → ^6^H_5/2_ transition^52^. The observed emission is likely due to host-related defects or charge transfer processes rather than direct La³⁺ luminescence. Lanthanum (La³⁺) has no 4*f* electrons, so it lacks the characteristic *f*-*f* transitions of other rare earth ions^53^. The spectrum out of the Nd doped sample displays a broad characteristic emission peak around 448 nm with two additional transitions at 560 and 636 nm. These emissions have been reported to be attributed to the ^4^G_7/2_→^4^I_9/2_ and to the ^4^G_5/2_→^4^I_9/2_ transitions, respectively^54^. The emission intensity of the Nd doped glass is slightly higher than that of B-Na-La glass. The broadness of the peak suggests matrix influence or overlapping transitions. The gadolinium-doped glass (B-Na-Gd) shows the demonstrates highest prominent emission peak (greater than 2000 a.u) centered at 450 nm, and a secondary one peak at 580 nm arising from the^5^D_0_→^7^F_0_ transition^55^. The emission peak at 580 nm may resulted from host sensitization or defect-related luminescence enhanced by Gd doping. The Ho-doped glass also exhibits a broad emission band peaking around 443 nm, and a secondary transition at 585 nm corresponding to the^5^S_2_→^5^I_8_ transition^56^. Holmium (Ho³⁺) typically showed visible emissions from transitions such as ⁵F₃ → ⁵I₈ or ⁵S₂ → ⁵I₈ ^9^. The broad nature of the emission suggests significant interaction with the glass matrix. For the Er-doped glass, the spectrum also displays a sharp emission band around 450 nm with intensity slightly below 700 a.u. The observed peak centered at 577 nm may be attributed to the ^4^S_3/2_→^4^I_15/2_ transition^52^. Finally, the ytterbium-doped glass shows a nearly weak, emission centered at 445 nm with intensity below 400 a.u, and a secondary broad peak centered at 580 nm. The peak at 580 nm is corresponding to the ^4^F_5/2_ → ^4^F_7/2_ transition in Yb^3+^ ions [9]. Yb³⁺ primarily emits in the near-infrared ^2^F_5/2_ → ^2^F_7/2_ at ~ 980–1030 nm, so the visible emission is mostly due to host-related defects or inefficient excitation at λ_ex_ = 220 nm^57^.

The broad emission bands observed in all spectra, including the undoped glass, suggest that the glass matrix contributes to the luminescence, likely through defect states such as oxygen vacancies or boron-oxygen structural units^51,58^. The addition of rare earth ions enhances this emission or introduces characteristic *f*-*f* transitions. The 220 nm excitation wavelength likely excites the host lattice. The energy is then transferred to the rare earth ions, leading to their characteristic emissions. This process, known as sensitized luminescence. This process particularly, found to be more efficient for Gd³⁺, as evidenced by its intense emission^53^. The exceptional intensity of the Gd-doped glass is due to the efficient population of the ⁶P₇_/_₂ level via the 220 nm excitation. The differences in emission intensity and peak positions reflect the unique electronic structures of each rare earth ion. For example, Gd³⁺ (4*f*⁷) has a stable half-filled 4*f* shell, leading to intense UV emission, whereas Yb³⁺ (4*f*¹³) has limited visible emission due to its primary near-infrared transition^9^. The moderate emission intensities observed in rare earth-doped glasses (e.g., Ho, Er, Nd) indicate a combination of *f*-*f* transitions and host-related luminescence. The amorphous nature of the glass matrix causes inhomogeneous broadening of the transitions caused by rare earth ions, which are typical in borate-based glasses^9^. The weak visible emission from Yb³⁺ and La³⁺ is expected, given their electronic configurations and primary emission ranges.

#### Color chromaticity coordinates

**Fig. 2:**
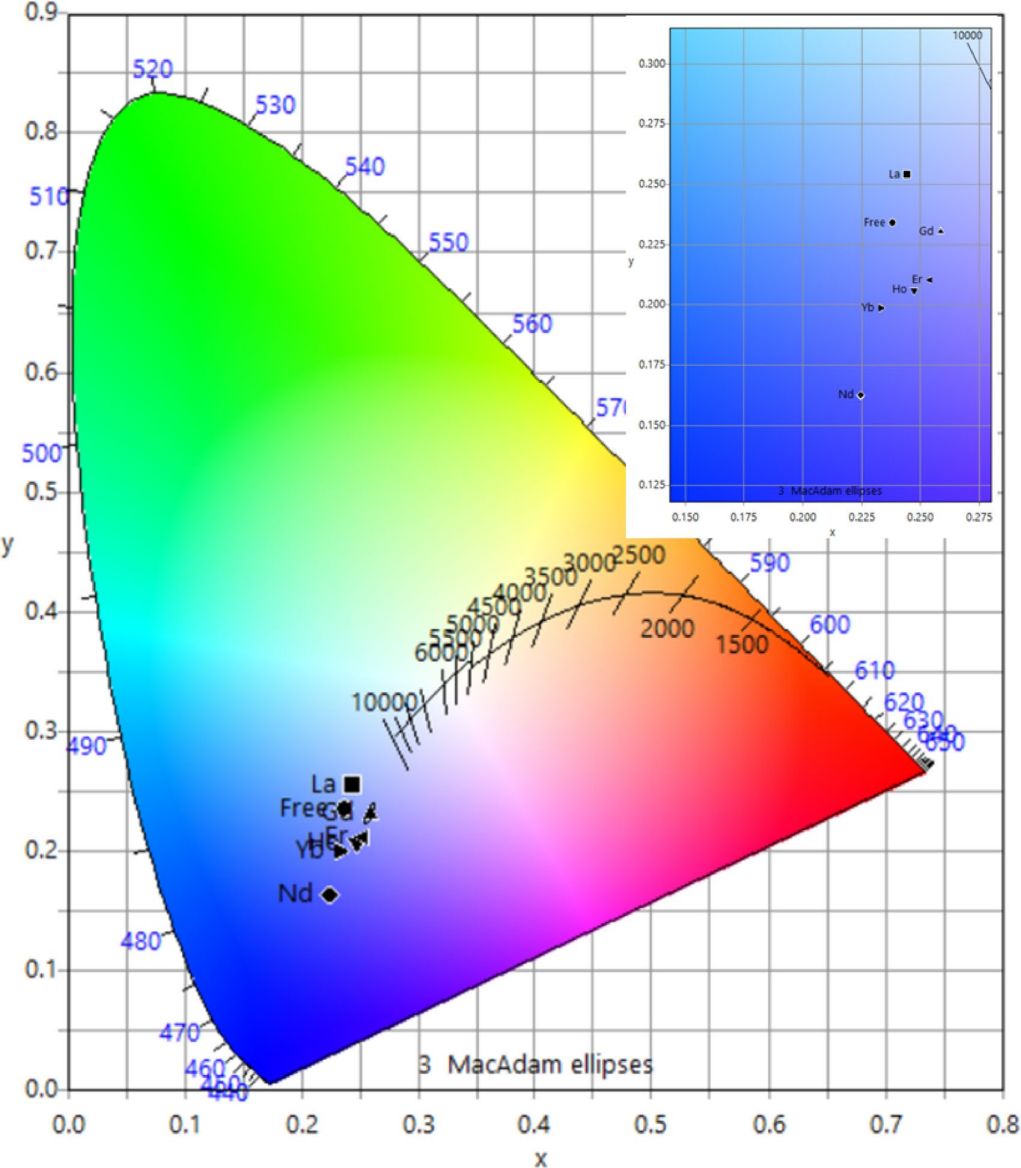
The CIE diagram of the prepared glasses.

To characterize the emission color of glasses, the standard Commission International de l’Eclairage (CIE) 1931 chromaticity diagram is applied. From the luminescence spectra, the chromaticity coordinates of specimens were calculated using color calculator software SpectraChroma (CIE coordinate calculator). The CIE diagram of the prepared glasses is shown in Fig. 2. All the glasses are found to fall mainly in the blue region and ranging from light to deep blue, with a slight deviation of the Gd, Ho and Er doped samples towards the violet region. The obtained values of the color chromaticity coordinates (*x*, *y*) are listed in Table 4. Important color properties should be discussed here; they are the color purity %, the luminous effectiveness of radiation (*LER*) and the correlated color temperature (*CCT*). The color purity% of the investigated glasses is calculated according to the formula^59^:

**Table 4:** CIE chromaticity coordinates, correlated color temperature (*CCT*, K) and luminous effectiveness of radiation (*LER*).

Sample code	Color coordinates (x, y)	CCT (K)	Color purity %	LER (lm/W_em_)
B-Na	0.238, 0.234	35,630	32.39	181
B-Na-La	0.244, 0.254	21,240	26.99	219
B-Na-Nd	0.225, 0.162	7617	51.97	126
B-Na-Gd	0.259, 0.230	28,460	29.6	187
B-Na-Ho	0.247, 0.206	127,608	37.46	155
B-Na-Er	0.254, 0.21	77,582	35.39	169
B-Na-Yb	0.233, 0.199	455,330	41.39	165

11 $$\:Color\:purity\%=\frac{\sqrt{{\left(x-{x}_{i}\right)}^{2}+{\left({x}_{d}-{x}_{i}\right)}^{2}}}{\sqrt{{\left(y-{y}_{i}\right)}^{2}+{\left({y}_{d}-{y}_{i}\right)}^{2}}}\times\:100\%$$

where (*x*, *y*) is the color coordinate of the samples, (*x*_*i*_, *y*_*i*_) is the CIE of an equal-energy illuminant, and (*x*_*d*_, *y*_*d*_) is the coordinate corresponding to the peak of the dominant wavelength. The color purity% of the investigated glasses is calculated using the D93 standard illuminants of white light (0.283, 0.297) and tabulated in Table 4. The color purity values are found to vary from 26.99 to 51.97 depending on the type of the RE dopant material. Low *LER* magnitudes around 169 lm/W_em_ are discovered to reveal the studied glasses (see Table 4). Finally, the correlated color temperature (*CCT*) is calculated by the McCamy empirical formula^60^:

12 $$CCT = - 449n^{3} + 3525n^{2} - 6823n + 5520.33$$

where $\:n=(x-{x}_{e})/\left(y-{y}_{e}\right)\:$; $\:{x}_{e}=0․332\:\mathrm{a}\mathrm{n}\mathrm{d}\:{y}_{e}=0․186\:$are the epicenter of convergence. The blank glass sample B-Na exhibits a high *CCT* value (35630 K), suggesting emission in the blue to near-ultraviolet (UV) spectrum. This behavior is consistent with the intrinsic properties of borate glasses, which are known for their transparency in the UV-visible region and their ability to host various dopants without significant alteration of the glass network structure. The high *CCT* can be attributed to the presence of non-bridging oxygens (NBOs) in the borate network, which create localized states that facilitate blue emission (see Sect. 3.6.1). This characteristic makes undoped borate glasses suitable for applications in the UV-transmitting optical components and substrates for UV and blue light-emitting devices^61^. The La³⁺-doped sodium borate glass exhibits a lower *CCT* value (21240 K) compared to the undoped glass (35630 K), indicating a shift toward warmer white emission. Interestingly, this sample also showed a higher optical band gap^25^, which can be attributed to the role of La³⁺ as a network modifier. Unlike other RE³⁺ ions, La³⁺ has an empty 4*f* shell and does not participate in radiative transitions, but it influences the glass structure by reducing the number of NBOs and enhancing network connectivity^11,53^. This leads to wider band gaps and reduced defect-related emission, resulting in a red-shifted luminescence profile and hence a lower *CCT*^11,12^. These characteristics make La³⁺-doped borate glasses suitable for warm white lighting, optical coatings, and as host matrices for co-doping with activator ions in photonic devices^62^. The introduction of Nd^3+^ significantly lowered the *CCT* to 7617 K, corresponding to north sky daylight. Nd^3+^ ions are known for their sharp absorption bands in the visible region and strong near-infrared (NIR) emissions due to 4*f*-4*f* electronic transitions^63^. The observed decrease in *CCT* is attributed to the dominant emission from Nd³⁺ ions. This property renders Nd^3+^-doped glasses suitable for laser applications, especially in solid-state lasers and optical amplifiers operating in the NIR region^64^. Doping the B-Na glass with Gd^3+^ ions resulted in a *CCT* of 28,460 K, closely resembling that of the undoped glass. Gd³⁺ ions have a half-filled 4*f* shell, leading to relatively weak *f*-*f* transitions^65^. However, Gd^3+^ ions possess a high atomic number, enhancing the glass’s ability to attenuate high-energy radiation^66^. This characteristic makes Gd^3+^-doped glasses promising candidates for radiation shielding materials and scintillators in medical imaging and nuclear applications^67,68^. Doping with Ho_2_O_3_ led to a remarkable high *CCT* of 127,608 K, suggesting emission in the UV region. Ho^3+^ ions exhibit multiple energy levels that facilitate upconversion processes, where two or more low-energy photons are absorbed, and a single high-energy photon is emitted (second harmonic generation)^69^. The observed high CCT in B-Na-Ho glasses is likely due to such upconversion mechanisms, resulting in UV emission in which make it a potential candidate for applications requiring UV light sources, including photolithography, sterilization, and fluorescence microscopy^70,71^. Er_2_O_3_ doping is found to increase the *CCT* to 77,582 K, indicating a little more shifting towards the UV spectrum. Er^3+^ ions are well-known for their efficient upconversion capabilities, where infrared excitation leads to visible or UV emission^72^. The observed high *CCT* can be attributed to these upconversion processes, making Er³⁺-doped glasses suitable for applications in UV lasers, display technologies, and optical communication systems that utilize UV wavelengths^73,74^. Finally, Yb^3+^ ions primarily exhibit a simple energy level scheme^75^. The incorporation of Yb_2_O_3_ resulted in an ultra-high *CCT* of 455,330 K, implying emission in the extreme UV. The extraordinarily high *CCT*, in addition the *LER* and *purity%* values suggest the complex interactions within the glass matrix, possibly involving cooperative processes or energy transfer mechanisms leading to high-energy photon emission. Consequently Yb^3+^-doped glasses are of interest for applications in high-energy photonics, including UV curing, sterilization, and as potential sources for UV lithography^76,77^.

### Dc electrical conductivity

**Fig. 3:**
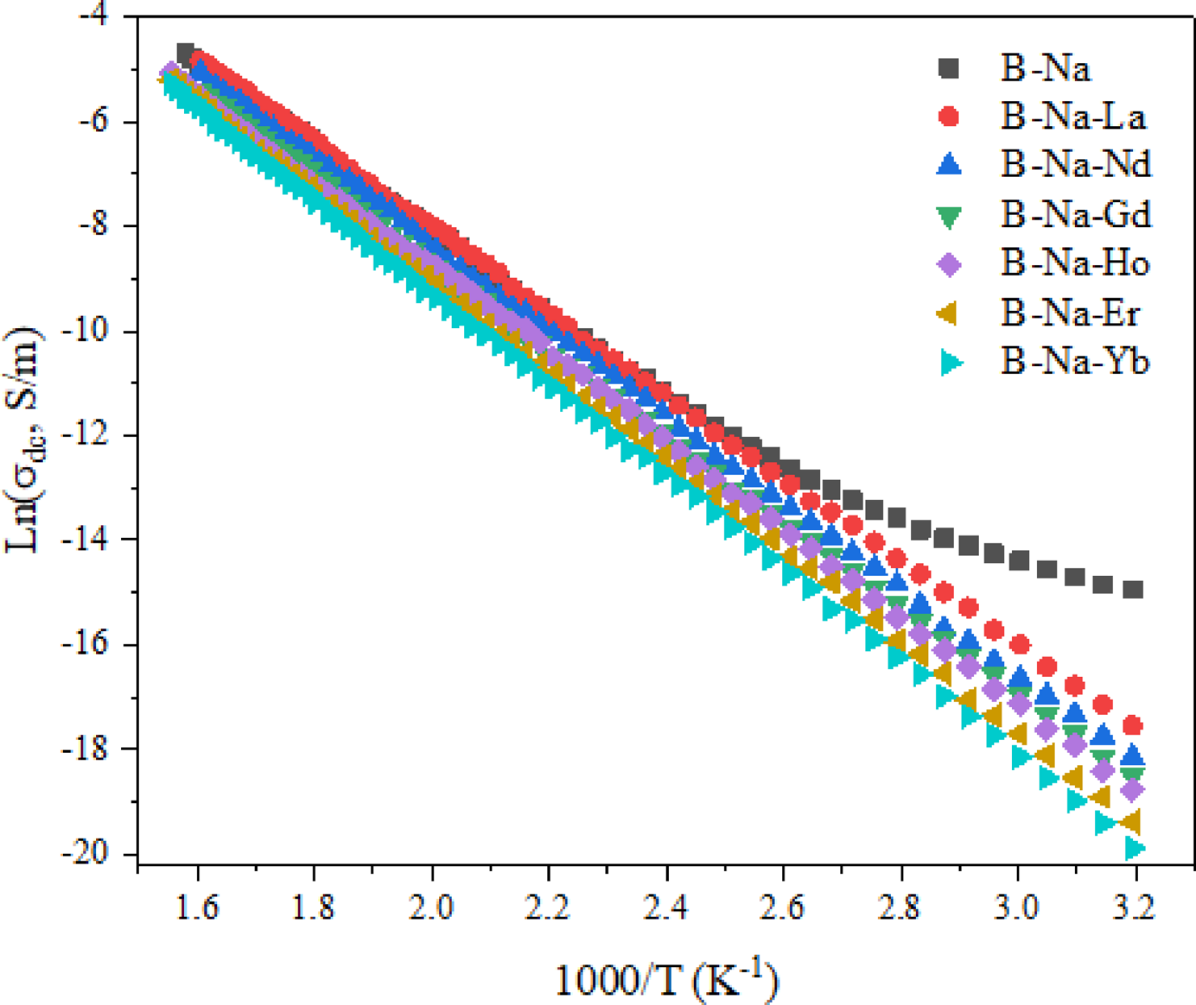
Temperature dependence of the dc conductivity of the studied glasses.

The measured dc conductivity (*σ*_*dc*_) for the studied glasses is found to exhibit a progressive rise from 1.68 × 10⁻⁸ to 3 × 10⁻² S/m as the temperature increases. This behavior is typically the behavior of temperature dependence of semiconductor. In semiconductors, the thermally activated *σ*_*dc*_ can be described by the following Arrhenius relation^78^:

13 $$\:{\sigma\:}_{dc}={\sigma\:}_{o\:}exp\left(\frac{-\varDelta\:E}{{k}_{B}T}\right)$$

where *σ*_*o*_ is the pre exponential value, *ΔЕ* is the activation energy, *k*_*B*_ is the Boltzmann constant and *T* is the absolute temperature. Figure 3 illustrates the variation *ln*(*σ*_*dc*_*)* as a function of (*1000/T*) for the investigated glasses in the temperature range 313–643 K. The dc conductivity profiles depicted in Fig. 3 generally exhibit a nearly linear Arrhenius-type trend, with minor deviations observed below approximately 370 K for the RE-free sample only. Furthermore, a consistent decline in *σ*_*dc*_ is observed as the ionic radius of the incorporated RE-ions decreases in the glasses. This behavior suggests that the dominant mechanism of *σ*_*dc*_ across the studied temperature range is due to the migration of Na⁺ ions (ionic conduction), supported by a limited effect of the 4*f* electronic transitions contributed by RE ions. This mixed ionic-electronic transition may require a relatively moderate activation energy (*ΔE*) to contribute to the conduction^79^. The magnitude of *ΔЕ* is computed for all samples and tabulated in Table 5. The observed increase in *ΔE* values with decreasing the ionic radius of RE ions is primarily attributed to enhanced structural rigidity and reduced ionic mobility within the glass matrix. It is worth noting that the field strength magnitudes of the studied glasses were calculated and presented in our previous work^25^, and they are found to decrease continuously with the decrease of RE ions (in the direction from La to Yb). This in turn may promote stronger RE–O bonds and increase the cross-linking of the glass network, which may reduce the free volume and hampers Na⁺ ion migration^11^. The stronger electrostatic interactions introduced by smaller RE ions also create deeper potential wells that require more thermal energy for Na⁺ ion activation^11,80^. This decrease in conductivity can also be attributed to the progressive filling of the 4f orbitals from La³⁺ (4*f*^0^) to Yb³⁺ (4*f*^13^), which enhances electron localization and shielding of the 5d–6s conduction states. The increased localization reduces the overlap between RE–O orbitals, thereby lowering carrier mobility and so the overall conductivity of the glass network^11^. These combined effects reduce ionic and/or electronic mobility and raise the energy required for charge transportation.

**Table 5:** The values of the activation energy (*ΔE*), the hopping distance (*R*), the defect concentration (*N*), the activation energy *W(R*_*p*_*)* and the dielectric constant (*ε*) for studied glasses.

Sample code	ΔE (eV)	*R*(nm)	*N*×10^20^(cm^− 3^)	W(*R*_*p*_) (eV)	ε
B-Na	0.692	1.47	2.00	0.621	1.66
B-Na-La	0.693	1.50	1.90	0.712	1.655
B-Na-Nd	0.709	1.54	1.76	0.720	1.63
B-Na-Gd	0.712	1.63	1.49	0.737	1.565
B-Na-Ho	0.717	1.78	1.14	0.738	1.50
B-Na-Er	0.738	1.94	0.88	0.747	1.462
B-Na-Yb	0.762	2.01	0.79	0.751	1.435

In a more refined theoretical perspective, it has been proposed that charge transport between localized states can occur via a multi-phonon hopping mechanism (MPH), particularly under conditions of weak coupling between charge carriers and the surrounding lattice. This conduction type involves thermally assisted carrier hopping across potential barriers separating defect-rich regions. The corresponding hopping rate, which reflects the frequency of such transitions, is quantitatively expressed through a formulation that accounts for the influence of multiple phonon interactions on tunneling dynamics, as originally discussed by Shimakawa and Miyake^81,82^:

14 $$\:\varGamma\:={\nu\:}_{0}{exp}\left(-\gamma\:p\right){[1-exp(-h{\nu\:}_{0}/{k}_{B}T\left)\right]}^{-p}$$

where $\:\gamma\:=\left[{ln}\left(\frac{\varDelta\:}{{E}_{m}}\right)-1\right]$, $\:p=\varDelta\:/h{\nu\:}_{0}$, *h* is Planck constant, *Δ* is the site separation energy, $\:{\nu\:}_{0}\sim{10}^{13}\:Hz$ is the optical phonon frequency and *E*_*m*_ is the electron-lattice coupling energy. Under the condition of high phonon population ($\:h\nu\:\ll\:{k}_{B}T$), electrical conductivity is predominantly governed by multi-phonon hopping^83^. This mechanism necessitates both the absorption and emission of *p* phonons in order to bridge the energy gap between localized sites. The corresponding hopping rate, *Γ*, scales with the Bose-Einstein distribution raised to the power *p*. Notably, the rates of phonon absorption and emission are equal, and both vary as $\:{(h{\nu\:}_{0}/{k}_{B}T)}^{p}$.

**Fig. 4:**
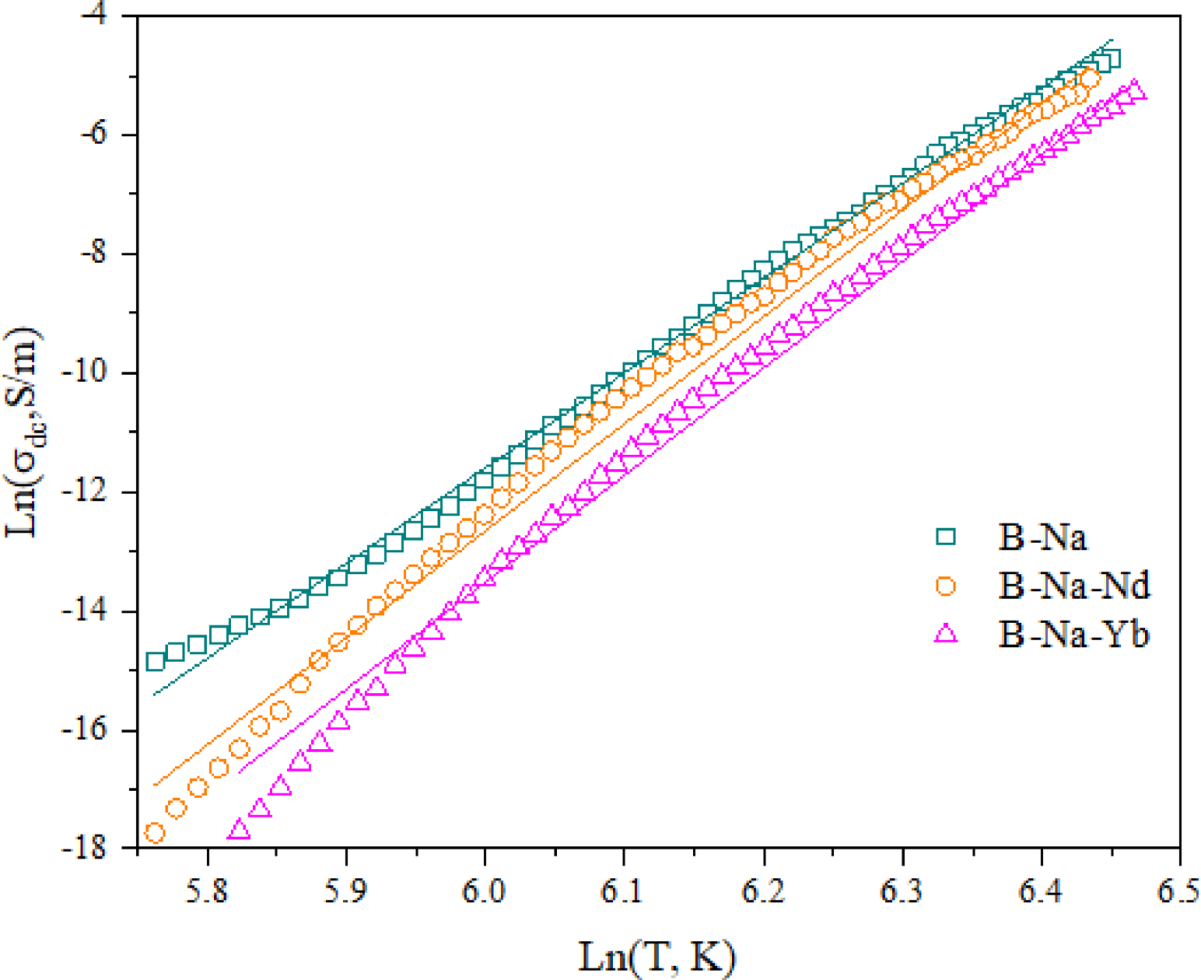
The relation between ln(σ_dc_) and ln(T) for B-Na, B-Na-Nd and B-Na-Yb samples, the open characters represent the experimental data while the solid lines represent the fitting according to the MPH approach.

The conductivity, *σ*_*dc*_, associated with the MPH rate can be expressed through the following mathematical relation^84^:

15 $$\:{\sigma\:}_{dc}={N}_{c}{\left(eR\right)}^{2}\varGamma\:/6{k}_{B}T\:\propto\:{T}^{p}$$

where *N*_*c*_ is the number of localized carriers, *e* is the charge of electron and *R* is the hopping distance. The experimental and theoretical values of dc conductivity (*σ*_*ex.*_ and *σ*_*th.*_), calculated using Eq. 15, are summarized in Table 6 for all studied glass samples at different temperatures. Figure 4 shows the relation between of *ln*(*σ*_*dc*_*)* against *ln(T)* for B-Na, B-Na-Nd and B-Na-Yb glass samples as examples, the open characters represent the experimental data while the solid lines represent the theoretical fitting according to Eq. 15. The obtained fitting to the experimental data confirms that *σ*_*dc*_ is proportional to *T*^*P*^ with *p* in the range of 16 to 18. A comparable values of *p* are reported by other researchers^81,85^ for different glass systems. Other fitted parameters for the glass samples under investigation are found as follows: *γ* is found to vary between 4.625 and 5, *E*_*m*_ showed a continuous decrease from 2.39 to 1.85 µeV. The values of *R* are presented in Table 5. *R* is shown to increase from 1.47 to 2.01 nm, which may associate to the conversion of BO₄ to BO₃ units, as well as to the resulting formation of non-bridging oxygens (NBOs), leading to a more open glass network^12^. The decrease of *N*_*c*_ suggests that RE ions act as deep traps, reducing the number of mobile charge carriers particularly for Ho³⁺, Er³⁺, and Yb³⁺, which have complex *f*-electron configurations^11^.

**Table 6:** Experimental (*σ*_*ex.*_ ) and theoretical (*σ*_*th.*_) Dc conductivity values for studied glass samples at different temperatures.

T(K)	B-Na		B-Na-La		B-Na-Nd		B-Na-Gd		B-Na-Ho		B-Na-Er		B-Na-Yb	
	*σ*_*ex*_.(S/m)	*σ*_*th.*_(S/m)	*σ*_*ex*_.(S/m)	*σ*_*th.*_(S/m)	*σ*_*ex*_.(S/m)	*σ*_*th.*_(S/m)	*σ*_*ex*_.(S/m)	*σ*_*th.*_(S/m)	*σ*_*ex*_.(S/m)	*σ*_*th.*_(S/m)	*σ*_*ex*_.(S/m)	*σ*_*th.*_(S/m)	*σ*_*ex*_.(S/m)	*σ*_*th.*_(S/m)
333	3.96 ×10^− 7^	4.29 ×10^− 7^	1.21 ×10^− 7^	1.72 ×10^− 7^	6.66 ×10^− 8^	1.02 ×10^− 7^	6.32 ×10^− 8^	7.89 ×10^− 8^	8.76 ×10^− 8^	7.29 ×10^− 8^	8.76 ×10^− 8^	5.12 ×10^− 8^	8.76 ×10^− 8^	4.30 ×10^− 8^
383	2.42 ×10^− 6^	4.01 ×10^− 6^	2.15 ×10^− 6^	2.13 ×10^− 6^	1.53 ×10^− 6^	1.26 ×10^− 6^	1.07 ×10^− 6^	9.78 ×10^− 7^	2.77 ×10^− 6^	9.03 ×10^− 7^	2.77 ×10^− 6^	6.34 ×10^− 7^	2.77 ×10^− 6^	5.32 ×10^− 7^
433	2.55 ×10^− 5^	2.85 ×10^− 5^	2.61 ×10^− 5^	1.93 ×10^− 5^	1.91 ×10^− 5^	1.15 ×10^− 5^	7.45 ×10^− 6^	8.89 ×10^− 6^	1.22 ×10^− 5^	8.21 ×10^− 6^	1.22 ×10^− 5^	5.77 ×10^− 6^	1.22 ×10^− 5^	4.84 ×10^− 6^
483	1.87 ×10^− 4^	1.64 ×10^− 4^	2.05 ×10^− 4^	1.38 ×10^− 4^	1.47 ×10^− 4^	8.20 ×10^− 5^	3.01 ×10^− 5^	6.35 ×10^− 5^	3.83 ×10^− 5^	5.86 ×10^− 5^	3.83 ×10^− 5^	4.12 ×10^− 5^	3.83 ×10^− 5^	3.45 ×10^− 5^
533	7.26 ×10^− 4^	7.92 ×10^− 4^	6.68 ×10^− 4^	8.13 ×10^− 4^	6.99 ×10^− 4^	4.82 ×10^− 4^	1.48 ×10^− 4^	3.73 ×10^− 4^	3.35 ×10^− 4^	3.45 ×10^− 4^	3.35 ×10^− 4^	2.42 ×10^− 4^	3.35 ×10^− 4^	2.03 ×10^− 4^
583	4.35 ×10^− 3^	3.32 ×10^− 3^	3.76 ×10^− 3^	4.08 ×10^− 3^	3.27 ×10^− 3^	2.42 ×10^− 3^	3.49 ×10^− 3^	1.87 ×10^− 3^	1.78 ×10^− 3^	1.73 ×10^− 3^	1.78 ×10^− 3^	1.22 ×10^− 3^	1.78 ×10^− 3^	1.02 ×10^− 3^

Another treatment of the experimental data of *σ*_*dc*_ suggested by Pramanik et al.^86^ can be applied. In this model, the dc conductivity *σ*_*dc*_ may originate as a result of a bipolaron transportation within the glass matrix following what is called “the correlated barrier hopping (CBH) model”, as follows:

16 $$\:{\sigma\:}_{dc}={\sigma\:}_{0}\mathrm{e}\mathrm{x}\mathrm{p}[-W({R}_{p})/{k}_{B}T]$$

with.

17 $$\:{\sigma\:}_{o}=\frac{{g}_{o}{N}^{2}}{15}\left[{R}_{p}^{5}-{R}_{min}^{5}\right]\mathrm{exp}\left(\frac{{W}_{M}}{{k}_{B}T}\right)$$

where W(R_p)_ is the activation energy, *g*_*o*_ is the density of states, *N* is the defect concentration, *R*_*p*_ is the critical percolation radius, *R*_*min*_ is a lower bound (cut-off) to the hopping distance and *W*_*M*_ is the hopping activation energy.

18 $$\:W\left({R}_{p}\right)={W}_{M}-\frac{2{e}^{2}}{\pi\:\epsilon\:{\epsilon\:}_{o}{R}_{p}}$$

where *ε* the dielectric constant, *ε*_*o*_ the free-space dielectric permittivity.

19 $$\:{g}_{o}=\frac{{e}^{2}}{4{k}_{B}T{\tau\:}_{o}\mathrm{exp}\left({W}_{M}/{k}_{B}T\right)}$$

where *τ*_*o*_ is associated with the period of the natural oscillation or resonance in systems subjected to alternating fields. *R*_*p*_ and *R*_*min*_ are given as follow:

20 $$\:{R}_{p}={\left(2․7\times\:\frac{3}{4\pi\:N}\right)}^{\raisebox{1ex}{$1$}\!\left/\:\!\raisebox{-1ex}{$3$}\right.}$$

21 $$\:{R}_{min}=\frac{2{e}^{2}}{\pi\:\epsilon\:{\epsilon\:}_{o}{W}_{M}}$$

**Fig. 5:**
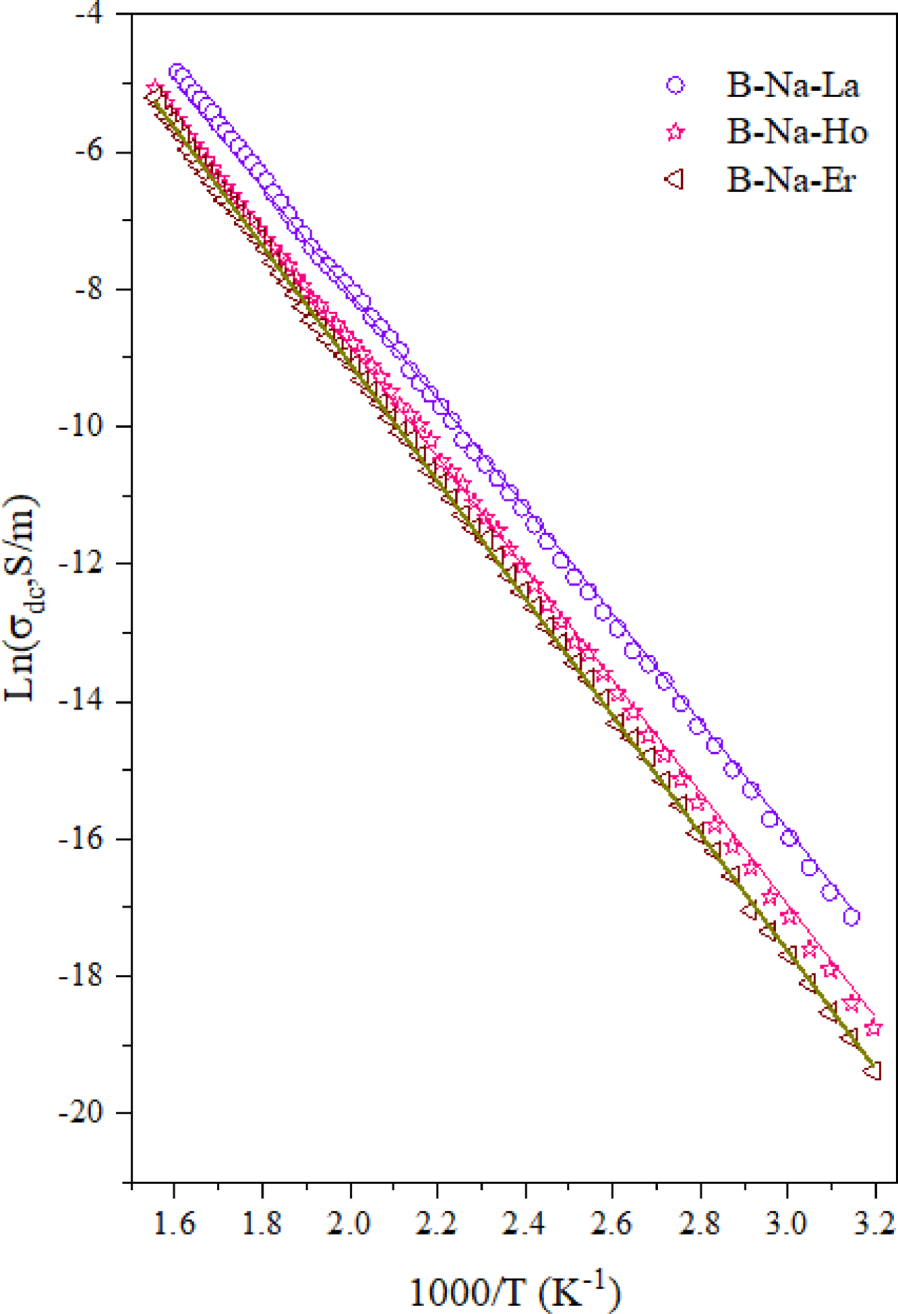
The variation of ln(σ_dc_) with (1000/T) for B-Na-La, B-Na-Ho and B-Na-Er glass samples, the open characters represent the experimental data while the solid lines represent the fitting according to CBH model.

Figure 5 presents *ln*(*σ*_*dc*_*)* as a function of *1000/T* for B-Na-La, B-Na-Ho and B-Na-Er glasses as examples. The solid lines represent fitting according to Eq. 16. Figure 5 revealed that the fitting of *σ*_*dc*_ for all the glasses are in good agreement with the experimental results over the whole temperature range, confirming the applicability of CBH mechanism for the studied glasses. Different parameters extracted from fitting the dc conductivity by the CBH model are found very close to those obtained from the fitting according to the multi-phonon hopping approach. The values of *N* used to calculate *R*_*p*_ and correspondingly *W(R*_*p*_*)* are presented in Table 5. The calculated values of *R*_*p*_ according to CBH model are found exactly similar to the obtained *R* values according to MPH approach. It is worth mentioning that the computed *W(R*_*p*_*)* values that displayed in Table 5 are close to values of thermal activation energy deduced from Eq. 13. Lastly, the obtained dielectric constant *ε* for the different glasses are also tabulated in Table 5. The value of *ε* is found to drop from 1.66 to 1.43 upon doping with RE in the order (La, Nd, Gd, Ho, Er and Yb) indicating the decrease of polarizability of the glass matrix. This is due to the more tightly bound structure and reduced free carrier content in RE-doped samples^87^.

### Molar magnetic susceptibility, *χ*_*m*_

**Fig. 6:**
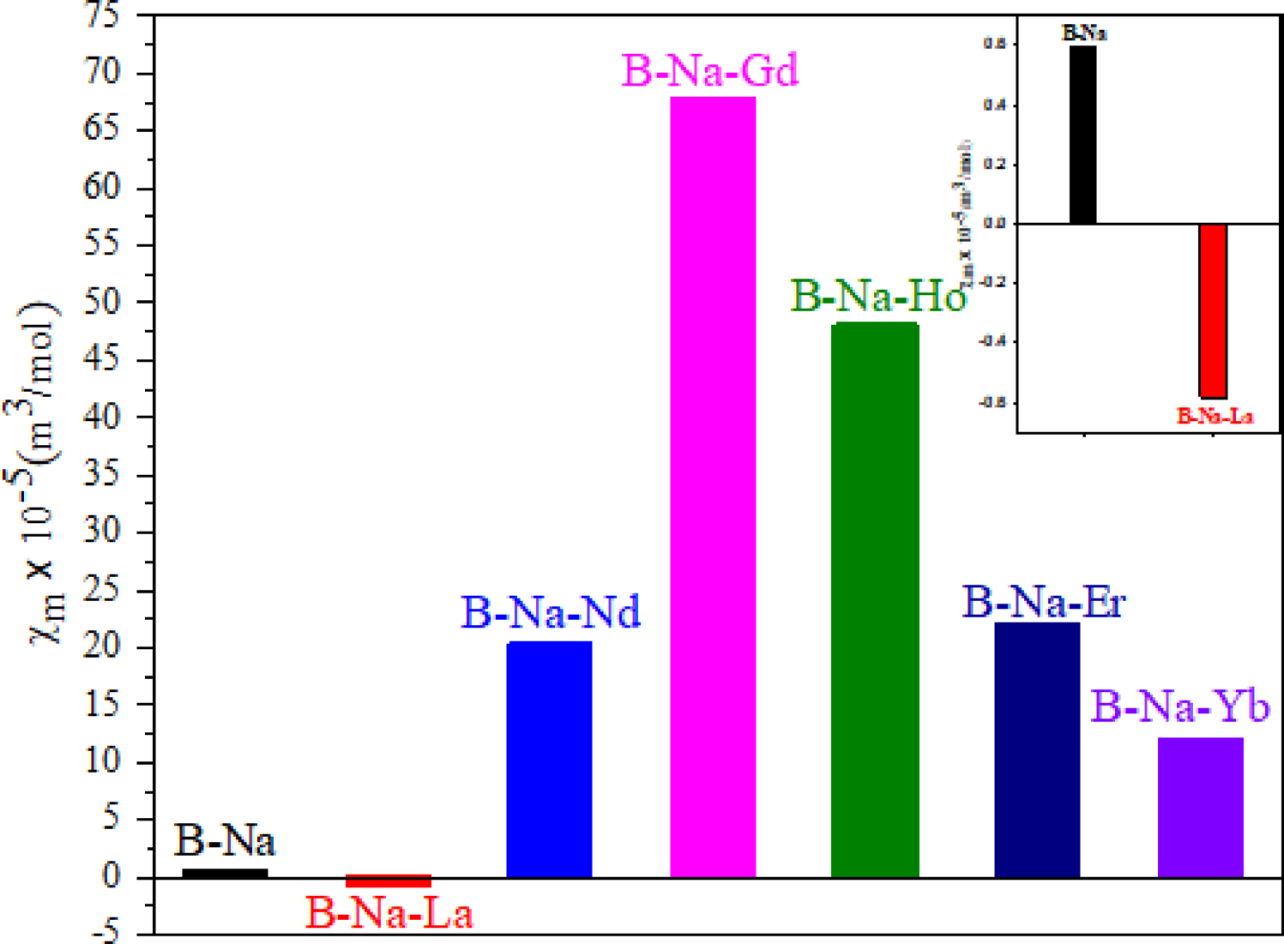
Molar magnetic susceptibility properties of the RE doped B-Na glass.

Gouy technique is used to explore the influence of the structural changes on the magnetic characteristics of the different solid materials. The magnetic susceptibility, *χ*_*m*_, is computed using the following Equation^88^:

22 $$\:{\chi\:}_{m}={\chi\:}_{g}{M}_{w}$$

where *χ*_*g*_ and *M*_*w*_ refer to the mass susceptibility and the molecular weight, respectively.

23 $$\:{\chi\:}_{g}=\frac{{C}_{bal}l(R-{R}_{o})}{{10}^{9}\:m}$$

where *C*_*bal*_ is balance calibration constant (*C*_*bal*_=1.35), *l* is sample length (cm), *R* is reading for tube plus sample, *R*_*o*_ is empty tube reading and *m* is sample mass (gm). The relation between *χ*_*m*_ and the studied glasses is shown in Fig. 6. Calculated values of *χ*^*m*^ show distinct behavior depending on the type of the doped RE ion. The B-Na glass exhibits a small positive *χ*^*m*^, indicating weak paramagnetic behavior likely arising from structural defects or NBO’s rather than intrinsic contributions from B³⁺ or Na⁺ ions^79,89^. Upon doping of B-Na glass with RE ions, the susceptibility increases significantly for Nd³⁺ and reaches the optimum value for the Gd³⁺ doped sample. After that, it goes to decrease for the Ho³⁺, Er³⁺ and Yb³⁺ doped glasses. This may be majorly due to the influence of the unpaired 4*f* electrons possessed by these RE dopants. Nd³⁺ (4*f*³) introduces three unpaired electrons leading to moderate paramagnetism, while Gd³⁺ (4*f*⁷) with a half-filled 4*f* shell shows strong paramagnetism owing to seven unpaired electrons^90,91^. Ho³⁺ (4*f*¹⁰) and Er³⁺ (4*f*¹¹) contribute significant magnetic moments through a combination of spin and orbital angular momentum effects^91,92^, while Yb³⁺ (4*f*¹³), with only one unpaired electron, results in weaker paramagnetism^91,93^. The only sample shown to present diamagnetic nature is the La³⁺-doped sample, which is may be accepted to be due to its 4*f*⁰ configuration with no unpaired electrons^11,94^. These magnetic properties may align with the known shielding behavior of 4*f* electrons and the minimal influence of the glassy matrix on the RE magnetic moments^95^.

### Thermal analysis

#### Differential scanning calorimetry (DSC)

**Fig. 7:**
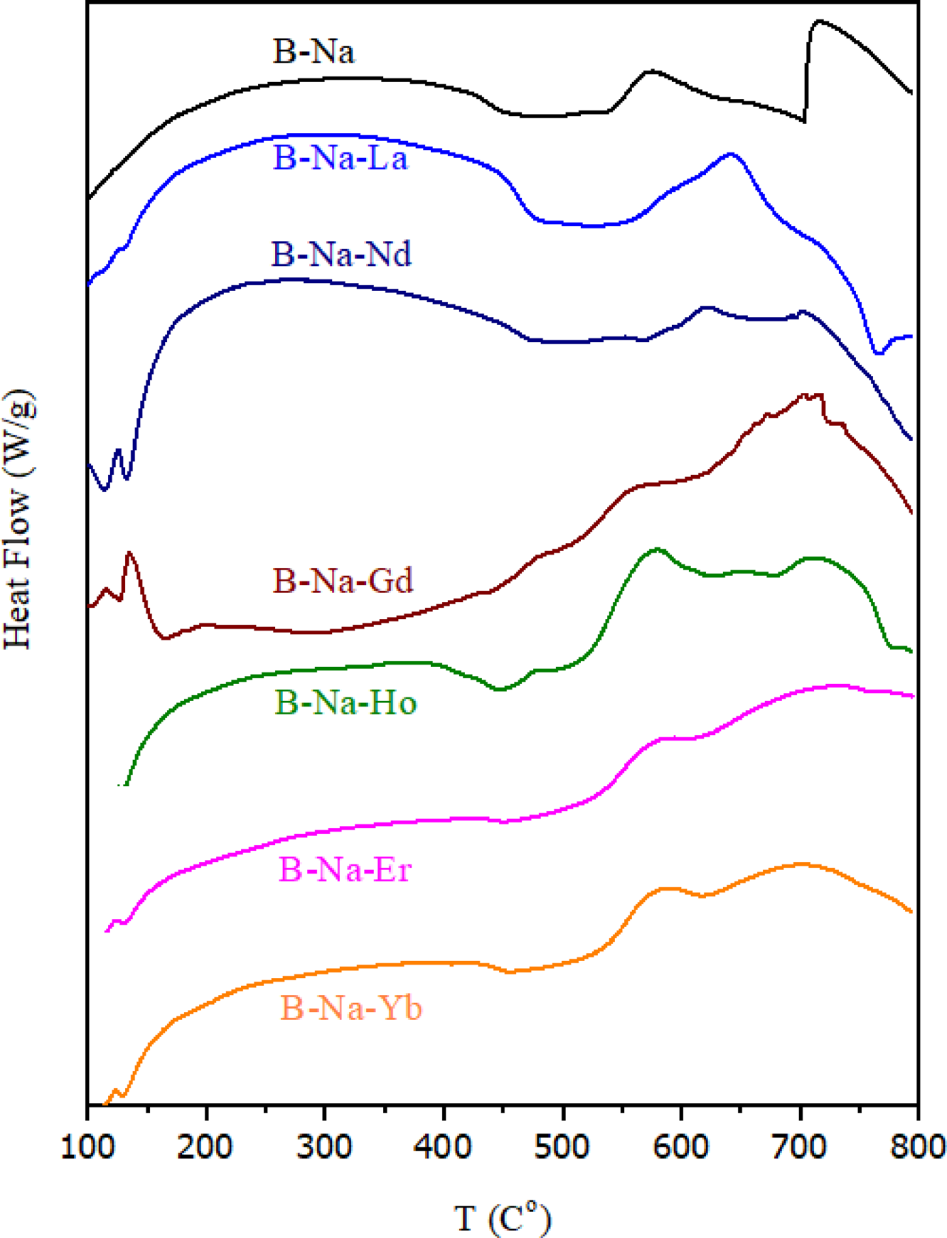
DSC thermograms of the studied glasses.

Differential scanning calorimetry (DSC) is employed to investigate the thermal behavior of the prepared glass samples. Figure 7 represents the DSC thermograms for the studied glasses. The thermal parameters, including glass transition temperature (*T*_*g*_), crystallization temperature (*T*_*c*_), peak of the crystallization (*T*_*p*_), and melting temperature (*T*_*m*_), are summarized in Table 7. It is noticed that some parameters cannot be identified clearly. These parameters are crucial for understanding the thermal stability of glasses. The base glass (B-Na) exhibited *T*_*g*_ value of 422 °C and *T*_*c*_ value of 536 °C. Incorporation of RE oxides resulted in variations in *T*_*g*_, with values ranging from 426 °C to 450 °C, depending on the type of the RE dopant. Notably, the Nd-containing sample displayed the highest *T*_*g*_ value of 450 °C, suggesting enhanced network rigidity due to stronger Nd–O bond interactions within the glass matrix. It is worth noting that the glasses doped with Nd and La ions are found to present the highest of the single bond strength values. Similar trends have been observed in previous studies, where the addition of RE ions increased *T*_*g*_, indicating a more rigid glass network^96^. The crystallization behavior varied also with the nature of the RE dopant. As follow; some doped glasses exhibited single crystallization exotherms, while others showed multiple exothermic peaks, indicating complex devitrification mechanisms. For instance, B-Na-Gd, B-Na-Ho and B-Na-Yb glasses demonstrate two *T*_*c*_ and two *T*_*p*_ values for each, suggesting overlapping or sequential crystallization events. The B-Na-Gd sample showed crystallization peaks at *T*_*c1*_ = 515 °C and *T*_*c2*_ = 623 °C, with associated *T*_*p*_ values at 560 °C and 703 °C, and the B-Na-Ho sample showed crystallization peaks at *T*_*c1*_ = 515 °C and *T*_*c2*_ = 683 °C, with associated *T*_*p*_ values at 580 °C and 711 °C, while B-Na-Yb sample showed crystallization peaks at *T*_*c1*_ = 540 °C and *T*_*c2*_ = 633 °C, with associated *T*_*p*_ values at 587 °C and 701 °C. This result confirms the possible formation of intermediate or metastable phases. Such behavior aligns with findings in similar glass systems, where RE doping influenced crystallization kinetics and phase formation^97^. For the *T*_*m*_ values, it can be shown in Fig. 7 that the measuring temperature range is not enough to detect the T_m_ values of all the studied glasses. *T*_*m*_ values are generally high across the recorded samples (~ 704–780 °C) with noticeable increase with the presence of RE-ions. It is reported that glasses doped with heavier rare earth elements demonstrated slightly elevated *T*_*m*_ compared with the rare earth free sample, which may be attributed to their higher atomic masses and corresponding influence on glass compactness and stability of the glass network^98^. Furthermore, the thermal stability of the glass samples, represented by *ΔT* and defined as $\:\varDelta\:T={T}_{c}-{T}_{g}$. Building on this parameter, M. Saad et al.^99^ proposed two additional criteria to effectively evaluating the thermal stability of glass systems. These parameters are:

**Table 7:** The values of the glass transition temperature (*T*_*g*_), the crystallization temperature (*T*_*c1*_ and *T*_*c2*_ ), the peak of the crystallization (*T*_*p1*_ and *T*_*p2*_ ), the melting temperature (*T*_*m*_), and the thermal stability parameters (*ΔT* ,*H*′ and *S*) for the B_2_O_3_-Na_2_O-RE_2_O_3_ glasses.

Sample	T_g_ (°C)	T_c1_ (°C)	T_c2_ (°C)	T_p1_ (°C)	T_p2_ (°C)	T_m_ (°C)	ΔT (°C)	H′	S (°C)
B-Na	422	536	–	574	–	704	114	0.270	10.27
B-Na-La	441	551	–	642	–	767	110	0.249	22.70
B-Na-Nd	450	570	–	622	–	-	120	0.267	13.87
B-Na-Gd	429	515	623	560	703	-	86	0.200	9.02
B-Na-Ho	426	515	683	580	711	780	89	0.209	13.58
B-Na-Er	440	527	-	576	-	-	87	0.198	9.69
B-Na-Yb	435	540	633	587	701	-	105	0.241	11.34

24 $$\:{H}^{{\prime\:}}=\frac{\varDelta\:T}{{T}_{g}}$$

and

25 $$\:S=({T}_{p}-{T}_{c}){H}^{{\prime\:}}$$

The values of *ΔT*, *H*′ and *S* are presented in Table 7. A larger *ΔT* implies a broader temperature interval between the onset of crystallization and the glass transition temperature, indicating higher thermal stability and resistance to devitrification. For instance, B-Na-Nd exhibited the highest *ΔT* ≈ 120°C, suggesting superior glass-forming ability (GFA)^100^. H′ accounts for relative stability by normalizing *ΔT* with respect to *T*_*g*_. This allows better comparison across different glass compositions. The highest observed *H’* ≈ 0.267 is found to exhibit for the B-Na-Nd glass, indicating a favorable balance between transition and crystallization temperatures^101,102^. The *S* parameter combines both the width of the stability range and the proximity of the crystallization and peak of crystallization temperatures. It gives a weighted measure of thermal robustness. The highest *S* value of about 22.7 is observed for the B-Na-La glass, reinforcing its suitability for thermally demanding applications^103^. Glasses with high *ΔT* and *H*′ are excellent candidates for optical amplifiers, waveguides, and lasers due to their structural integrity and resistance to devitrification. Nd³⁺ and Yb³⁺ doped glasses are widely used in solid-state lasers and amplifiers^104^. Glasses with high *S* value are suited for electronics as hermetic seals and encapsulation materials due to their thermal stability and structural rigidity^11^. Incorporation of rare earth ions like Ho³⁺ and Gd³⁺ imparts radiopacity and therapeutic effects, making these glasses attractive for biomedical imaging and implant applications. The high thermal stability ensures safety during sterilization and processing^105^. Finally, the variation in different investigated thermal parameters reflects changes in network connectivity, structural rearrangement, and thermal stability induced by the specific ionic radius and field strength of each RE³⁺ ion^11^.

#### Thermogravimetric analysis (TGA)

**Fig. 8:**
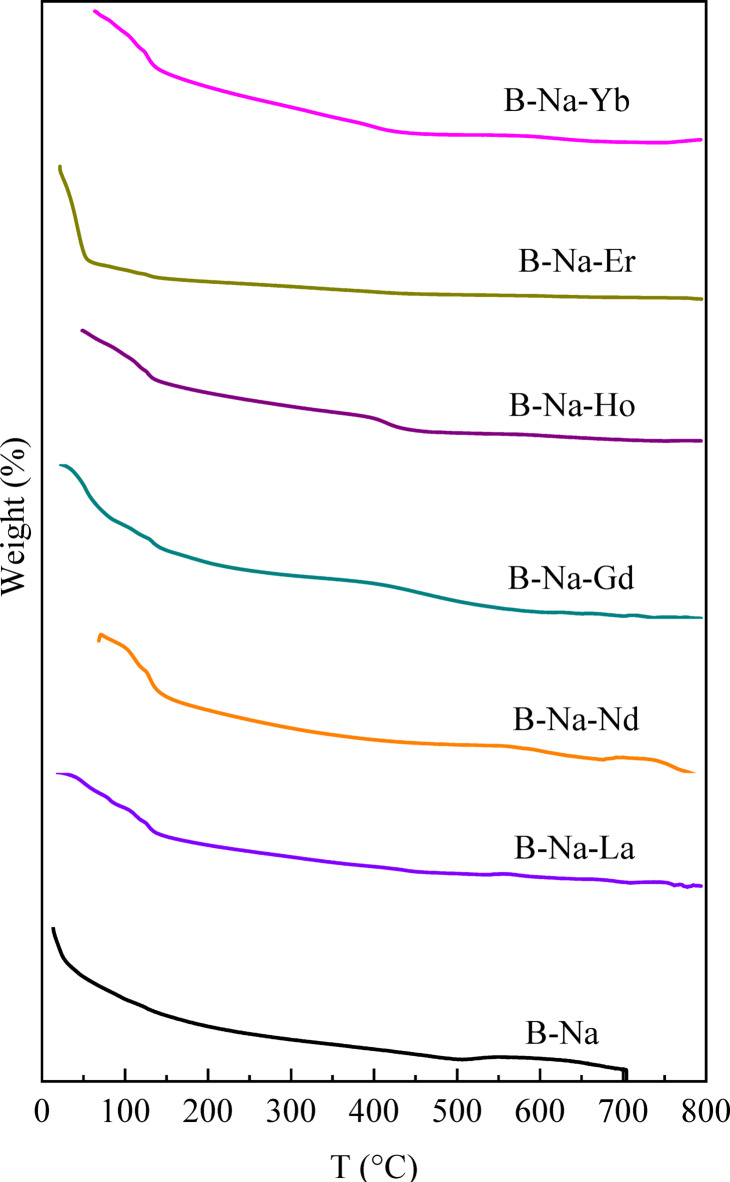
TGA thermograms of the studied glasses.

Thermogravimetric analysis (TGA) is conducted to assess the thermal stability and decomposition behavior of the glass samples. As can be shown in Fig. 8, all RE-doped compositions exhibited minimal mass loss (typically less than 1%) up to 800 °C, except the first stage of heating confirming the thermal robustness of the borate-based glass matrix. The initial weight loss observed below 200 °C is primarily attributed to the desorption of physically adsorbed water^106^. The RE-doped glasses displayed slightly varied thermal decomposition profiles. For example, B-Na-Nd glass showed marginally higher initial weight loss, possibly indicating higher hygroscopicity. However, no significant multi-step degradation patterns are observed, suggesting that the incorporation of RE₂O₃ does not compromise the bulk thermal stability of the glass system^107^. Only one maximal mass loss of about 2% is shown for the undoped B-Na glass. The observed mass loss is recorded at approximately 700 °C, where this temperature meets the T_m_ value recorded from the DSC curve in Fig. 7.

#### Thermal conductivity

Thermal conductivity (*ϕ*) is a key thermal property that reflects how readily heat can pass through a material. Materials that act as insulators such as glass, polymers, and ceramics typically exhibit thermal conductivity values in the range of approximately 0.1–2 W/m·K^108^. For the present study, the thermal conductivity of the glass samples under investigation are measured at room temperature using the following equation^109^:

26 $$\:Q=\varphi\:A\frac{{T}_{2}-{T}_{1}}{\varDelta\:x}$$

where *Q* is the equilibrium heat flow per second, *ϕ* is the experimental thermal conductivity, *A* and *Δx* are the surface area and thickness of the sample while, *T*_*2*_ and *T*_*1*_ are the temperature of the hot and cold surfaces, respectively. Conversely, in the case of semiconducting glasses, the parameter *ϕ* can be described using the following expression^109^:

27 $$\:\varphi\:=\:{\varphi\:}_{L}+{\varphi\:}_{e}+{\varphi\:}_{bp}$$

where *ϕ*_*L*_, *ϕ*_*e*_ and *ϕ*_*bp*_ are the contributions of lattice vibration, electron and bipolaron motion to the thermal conductivity, respectively. According to the Wiedemann-Franz law, the *ϕ*_*e*_ can be calculated for the present glass samples as follow^109^:

28 $$\:{\varphi\:}_{e}=\frac{1}{3}{\left(\frac{\pi\:{k}_{B}}{e}\right)}^{2}{\sigma\:}_{dc}T$$

Also, *ϕ*_*bp*_ is calculated by using the Equation^110^:

29 $$\:{\varphi\:}_{bp}=\frac{1}{4{e}^{2}}{\left(\frac{{E}_{g}}{T}+4{k}_{B}\right)}^{2}{\sigma\:}_{dc}T$$

where *E*_*g*_ is the band gap energy^25^. The values of *ϕ*, *ϕ*_*e*_, *ϕ*_*bp*_ and *ϕ*_*L*_ are listed in Table 8. The experimental values of *ϕ* of the investigated glasses show considerable variation depending on the RE dopant. As can be seen in Table 8, undoped B-Na glass exhibits the highest thermal conductivity (1.78 W/m·K), while the incorporation of RE elements generally leads to a decrease in *ϕ*. This decrease can be attributed to the probable increase of structural disorder occurred by the RE ions, which affects phonon transport mechanism in amorphous materials like glasses^11,111^. The analysis of total thermal conductivity into contributions from lattice vibrations (*ϕ*_*L*_), electronic conduction (*ϕ*_*e*_) and bipolaron motion (*ϕ*_*bp*_) provides deeper insight into the heat transport mechanisms. The dominance of *ϕ*_*L*_ in all samples emphasizes that phonons are the primary heat carriers, with minor contributions from electronic and bipolaronic mechanisms. This is consistent with the insulating nature of glass matrices, where free electron mobility is inherently low^11^. The values of *ϕ*_*L*_ are found to have approximately the same values of *ϕ*. Among the RE doped samples, B-Na-La has the highest *ϕ* and *ϕ*_*L*_ values of about 1.46 W/m·K suggesting that La³⁺ introduces the least disruption to the glass network. Conversely, B-Na-Gd shows the lowest thermal conductivity value ≈ 0.49 W/m·K, which can be linked to the enhanced scattering of phonons due to the mismatch in mass and size of the Gd³⁺ ion compared to the network formers. Such behavior aligns with the established understanding that RE dopants can introduce localized vibrational modes and structural inhomogeneities, thereby reducing thermal conductivity^112^. The low thermal conductivity values, particularly in Gd, Nd, and Yb-doped samples, suggest their suitability in thermoelectric materials, where minimizing heat transport while maintaining electrical conductivity is desirable^113^. Furthermore, the observed trends also highlight potential use in thermal barrier coatings and solid-state lighting, where control over thermal properties is crucial^114^.

**Table 8:** The values of the thermal conductivity (*ϕ*), the contributions of electron (*ϕ*_*e*_), bipolaron (*ϕ*_*bp*_ ) and lattice vibration (*ϕ*_*L*_ ) motion to the thermal conductivity for studied glasses.

Sample code	ϕ (W/m.K)	ϕ_e_ × 10^− 14^ (W/m.K)	ϕ_bp_ × 10^− 9^ (W/m.K)	ϕ_L_ (W/m.K)
B-Na	1.78	55.77	17.81	1.78
B-Na-La	1.46	4.68	1.54	1.46
B-Na-Nd	0.79	4.63	1.52	0.79
B-Na-Gd	0.49	2.89	0.94	0.49
B-Na-Ho	0.92	3.77	1.15	0.92
B-Na-Er	0.81	3.77	1.08	0.81
B-Na-Yb	0.77	3.77	1.26	0.77

## Conclusion

This study provides a comprehensive evaluation of the effects of rare-earth (RE) ion doping (La^3+^, Nd^3+^, Gd^3+^, Ho^3+^, Er^3+^, Yb^3+^) on the structural, optical, electrical, magnetic, and thermal properties of simple B-Na borate glass. The incorporation of 1 mol% RE oxides into the 50%B₂O₃–50%Na₂O glass matrix revealed several significant modifications across all measured parameters, which are directly correlated to the ionic radius, field strength, and electronic configuration of the RE dopants.

A remarkable increase in the molar refraction (*R*_*m*_) and molar polarizability (*α*_*m*_) upon doping with 1 mol% of RE ions. This enhancement is primarily attributed to the increased non-bridging oxygens (NBOs) and stronger local electric fields introduced by smaller, high field strength RE ions.

The significant increase of PL intensity for the RE doped samples, specially Gd^3+^ and Er^3+^ is achieved and is explained and confirmed by the high value of third-order nonlinear optical susceptibility (up to 1.76 × 10⁻¹² esu for Er^3+^) and the unique electronic structure of RE additives.

DC conductivity is found to decrease with decreasing RE ionic radii, due to the enhancement of crosslinking and the decrease of ion mobility. The DC conduction mechanisms consistent with both the multi-phonon hopping (MPH) and correlated barrier hopping (CBH) models. The increase of activation energy (*ΔE*) and the decrease number of mobile charge carriers suggest that RE ions act as deep traps. These findings reveal the tunability of electrical conduction through structural modification and highlight the suitability of these glasses for resistive switching and memory storage devices.

The paramagnetic behavior of all RE-doped samples (except La³⁺ doped glass) is consistent with the number of unpaired 4*f* electrons. Gd³⁺-doped glass showed the highest susceptibility due to its half-filled 4*f*⁷ configuration, making it suitable for magnetic sensor applications and potential radiation shielding due to high atomic number of Gd^3+^.

DSC measurements revealed an increase in the glass transition temperature (*T*_*g*_) upon RE doping, with the B-Na-Nd glass exhibiting the highest *T*_*g*_ (450 °C) and *ΔT* (~ 120 °C), indicating superior glass-forming ability. The presence of multiple crystallization peaks in Ho³⁺ and Yb³⁺-doped glasses indicates complex devitrification behavior. Such glasses are ideal candidates for optical waveguides, laser hosts, and thermally stable coatings.

The insulating nature increased upon doping B-Na glass with RE ions. When the thermal conductivity (*ϕ*) drops from 1.78 W/m·K for B-Na glass to 0.49 W/m·K for B-Na-Gd sample.

All the previous findings suggest RE ions as strong modifiers for various properties standing on the unique properties of every RE ion, moreover the variation of RE type with the same concentration leads to different modifications making a simple glass such as 50% B_2_O_3_-50% Na_2_O very tunable for several desired applications such as nonlinear optics, telecommunications, and optical switching for Er³⁺ and Ho³⁺ doped glass and photoluminescence devices, radiation shielding, solid-state lasers, UV-based technologies, lithography, sterilization tools, thermally robust hosts for optical amplifiers and encapsulation materials for Gd³⁺, Nd³⁺, Yb³⁺ and La³⁺ doped glass. Among all compositions, the Er³⁺-doped glass exhibits the highest nonlinear optical susceptibility (χ³ ≈ 1.76 × 10⁻¹² esu) and strong photoluminescent response, making it the most promising candidate for photonic and optical switching applications. Meanwhile, the Gd³⁺-doped glass shows superior PL intensity and low thermal conductivity (~ 0.49 W/m·K), indicating potential use in thermoelectric and radiation-shielding devices in addition to the photonics applications.

## Data Availability

The datasets generated during and/or analyzed during the current study are available from the corresponding author on reasonable request.
